# Functional mapping of the molluscan brain guided by synchrotron X-ray tomography

**DOI:** 10.1073/pnas.2422706122

**Published:** 2025-02-27

**Authors:** Michael Crossley, Anna Simon, Shashidhara Marathe, Christoph Rau, Arnd Roth, Vincenzo Marra, Kevin Staras

**Affiliations:** ^a^Department of Neuroscience, University of Sussex, Brighton BN1 9QG, United Kingdom; ^b^Wolfson Institute for Biomedical Research, University College London, London WC1E 6BT, United Kingdom; ^c^Diamond Light Source, Harwell Science and Innovation Campus, Didcot OX11 0DE, United Kingdom

**Keywords:** brain, synchrotron, X-ray tomography, neural circuit, feeding

## Abstract

Molluscan brains are composed of morphologically consistent and functionally interrogable neurons, offering rich opportunities for understanding how neural circuits drive behavior. Nonetheless, detailed component-level CNS maps are often lacking, total neuron numbers are unknown, and organizational principles remain poorly defined, limiting a full and systematic characterization of circuit operation. Here, we establish an accessible, generalizable approach, harnessing synchrotron X-ray tomography, to rapidly determine the three-dimensional structure of the multimillimeter-scale CNS of *Lymnaea*. Focusing on the feeding ganglia, we generate a full neuron-level reconstruction, revealing key design principles and revising cell count estimates upward threefold. Our atlas uncovers the superficial but also nonsuperficial ganglionic architecture, reveals the cell organization in normally hidden regions—ganglionic “dark sides”—and details features of single-neuron morphology, together guiding targeted follow-up functional investigation based on intracellular recordings. Using this approach, we identify three pivotal neuron classes: a command-like food-signaling cell type, a feeding central pattern generator interneuron, and a unique behavior-specific motoneuron, together significantly advancing understanding of the function of this classical control circuit. Combining our morphological and electrophysiological data, we also establish a functional CNS atlas in *Lymnaea* as a shared and scalable resource for the research community. Our approach enables the rapid construction of cell atlases in large-scale nervous systems, with key relevance to functional circuit interrogation in a diverse range of model organisms.

## Significance

Thanks to their accessible nervous systems, simple model organisms have provided some of the most fundamental insights into how neural circuits generate and control behavior. However, in many cases, understanding is constrained by the absence of detailed brain maps. Here, we address this challenge using a synchrotron source to enable rapid high-resolution X-ray imaging of whole multimillimeter-scale brains. Applied to the classic molluscan model, *Lymnaea*, we construct a detailed 3D atlas of its feeding circuit and use this to guide identification and functional characterization of pivotal cell types in the network. Our approach readily generalizes to CNS atlas-building in other model organisms enabling the interrogation and sharing of brain structures across research groups for comparative and functional studies.

How neural circuits generate complex behavior remains a key question in neuroscience. To comprehensively address this demands an experimental system in which behaviors are clearly defined and robustly expressed and where the underlying neuronal components are identified, quantified, and fully amenable to correlative functional characterization and causal manipulation. A variety of simpler systems, including crustaceans, mollusks, and annelids, have proved extraordinarily valuable for this purpose thanks to their large characteristic neurons and the accessibility of their nervous systems for functional interrogation, offering some of the most fundamental insights into mechanisms of pattern generation, neuromodulation, learning and memory, decision-making, and principles of circuit design (1–6). However, their benefits are often limited by the absence of comprehensive brain atlases that fully define the number, type, and arrangement of circuit components and thus inform a systematic functional characterization of network activity across the CNS. Although *tour de force* large-scale synaptic wiring maps based on 3D-electron microscopy methods are achievable in small nervous systems of choice organisms (7–10), notably in recent work using *Drosophila* (11–14), this approach is extraordinarily labor intensive and particularly challenging when applied to larger, multimillimeter-scale brains. It also significantly exceeds the requirements for an overview atlas where the aim is to inform detailed follow-up circuit-level electrophysiological investigation.

**Fig. 1.**
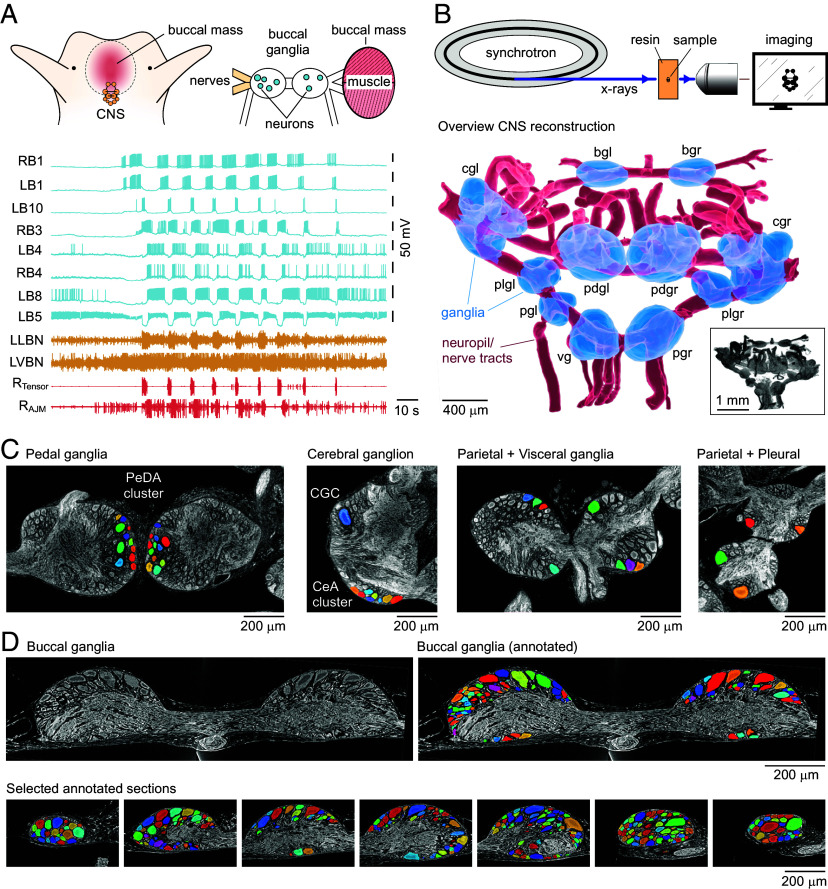
Synchrotron X-ray imaging of the molluscan brain. (*A*) (*Left*) Cartoon of *Lymnaea* head region with CNS indicated and (*Right*) preparation used for direct, simultaneous recordings of neurons, nerves, and muscles. (*Bottom*) Traces showing parallel intracellular recordings from eight neurons as well as extracellular recordings from two nerve bundles and two feeding muscles illustrate the exceptional accessibility of the system for circuit interrogation. Patterned activity corresponds to an internally generated fictive feeding rhythm (*B*) (*Top*) Experimental workflow. To capture whole-CNS maps, samples were embedded in Durcupan resin and imaged using synchrotron X-ray computed tomography (SXRT). (*Bottom*) Reconstruction based on whole-CNS SXRT dataset (2× objective), showing ganglionic volumes (blue) and principal nerve bundles (red). bg: buccal ganglia, cg: cerebral ganglia, pd: pedal ganglia, pl: pleural ganglia, pg: parietal ganglia, vg: visceral ganglion, l: left, r: right. The inset shows maximum projection of the raw image stack that was used to make the reconstruction. (*C*) Single section examples of structures in pedal, cerebral, parietal, visceral, and pleural ganglia from 2× objective dataset. A selection of annotated neurons including identified cell types (PeDA cluster neurons in pedal ganglia, CGC, and CeA cluster neurons in cerebrals), as well as other large neurons, are shown. (*D*) Central section (*Left*) with annotation (*Right*) of the paired buccal ganglia based on 10× objective dataset. (*Bottom*) Sample sections at different depths showing a fully annotated buccal ganglion with neurons labeled.

Here, we outline an accessible, generalizable methodological pipeline using synchrotron X-ray computed tomography (SXRT), a 3D volumetric imaging approach (15, 16), to enable rapid brain atlas-building and guide detailed investigation of circuit function in a simple model system. SXRT relies on the use of a powerful X-ray source to reveal micrometer-scale structural detail in samples that are many millimeters in size. We demonstrate its application in the molluscan model *Lymnaea stagnalis*, a leading simple system for probing fundamental mechanisms of circuit control thanks to its robust behaviors and experimental tractability (17–24). Over the last six decades, studies on *Lymnaea* have provided some of the most profound insights into neural control of central pattern generation, decision-making, perception, nervous system development, and learning and memory formation (18, 25–40). It is particularly well suited for CNS atlas-building studies because, like many invertebrate model organisms, its identified neurons are morphologically, positionally, and functionally consistent from brain to brain. As such, an accurate map can be generated that is applicable to all individuals, providing a key resource for aiding the reliable identification and targeting of defined cells. Moreover, simultaneous intracellular, extracellular, and muscle recordings across many channels are readily achievable (Fig. 1*A*), permitting a forensic investigation of the links between firing activity, intrinsic properties, connectivity, and motor output, information which can then be appended to morphological detail to provide a functional atlas.

Using SXRT, here, we imaged the whole *Lymnaea* brain to generate a 3D overview map that captures the organization of the main ganglia and principal nerve tracts. We then carried out a full detailed reconstruction of the paired buccal ganglia housing the main feeding circuitry including the ingestion and egestion central pattern generator networks. Our approach allowed us to revise the total neuron count upward more than three-fold compared to previous estimates, reveal key architectural principles that relate neuron volume to three-dimensional organization, and provide a comprehensive description of the cell population contained fully inside the ganglionic volume. As proof of principle, we then used our detailed cell atlas to identify and functionally characterize three neuron types in the brain: a command-like cell, a class of feeding central pattern–generating interneuron, and a unique behavior-specific motoneuron, together significantly updating our understanding of one of the already best-described neural control circuits. We also used our detailed maps, along with follow-up electrophysiological interrogation, to establish the foundations of a functional CNS atlas in *Lymnaea*, intended as an open-access, scalable resource for the research community. Our approach provides a pipeline to rapidly map multimillimeter-scale brain regions that can readily generalize to CNS atlas-building in other model organisms and thus enable the sharing of accurate representations of neuroanatomy across research groups to aid comparative studies and functional characterization.

## Results

### Organizational Principles of the Molluscan Brain Revealed using SXRT.

**Fig. 2.**
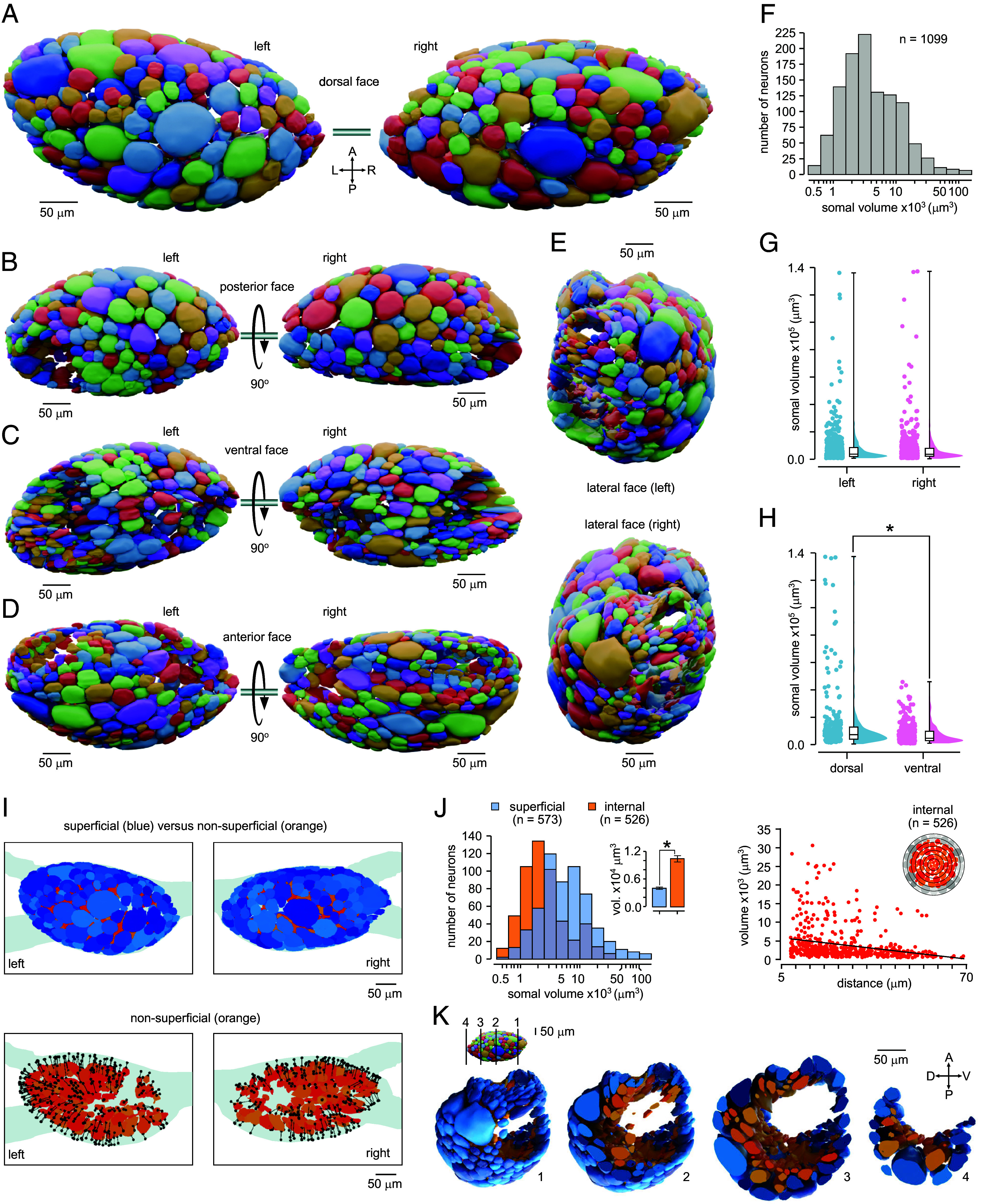
A complete three-dimensional model of the *Lymnaea* feeding ganglia. (*A*) Reconstruction from 10x image stacks of the paired (*Left* and *Right*) buccal ganglia showing the dorsal face with cell bodies labeled using a randomly assigned color palette. A: anterior, P: posterior, L: left, R: right. (*B*–*E*) Alternative faces of the ganglia show their structural organization and heterogeneity. (*F*) Histogram illustrating the range of volumes of cell bodies across the total population. (*G* and *H*) Cell volumes are not significantly different between *Left* and *Right* ganglia (*Left*, n = 542, *Right*, n = 557, Mann–Whitney, *P* = 0.97, U = 1.511) but (*H*) significantly larger on the dorsal compared to the ventral surface (dorsal, n = 256, ventral, n = 317, Mann–Whitney, *P* = 0.0001, U = 29940). (*I*) (*Top*) Reconstruction illustrating the superficial (blue) and internalized neuron population (orange/red) categorized based on distances from the ganglionic surface. (*Bottom*) Reconstruction showing internalized population alone with distance of each cell body from the surface indicated as a line and circle. (*J*) (*Left*) Overlapping histograms of volume distributions for superficial (blue) versus internalized (orange) populations. (Inset) Surface cell bodies are significantly larger than internalized ones (superficial, n = 573, blue bar, internalized, n = 526, orange bar, Mann–Whitney, *P* = 0.0001, U = 77869). (*Right*) Scatter plot showing cell body volumes versus distance from ganglionic surface for internalized cells. Cell body volumes decrease significantly with increasing depth (n = 526, Spearman’s, *P* = 0.0001, rs = −0.368). (*K*) Reconstructions showing the superficial (blue) and internalized (orange) cell populations in the right buccal ganglion at different lateral to medial depths (1–4) with positions indicated in the inset figure. A: anterior, P: posterior, D: dorsal, V: ventral.

To reveal the three-dimensional architecture of the *Lymnaea* CNS, we isolated, fixed, and processed whole brains from five adult animals and capsule-embedded them in Durcupan resin. Samples were then transferred to the Diamond Light Source national synchrotron facility, mounted in the X-ray path of the I13-2 beamline, and scanned (Fig. 1*B*). Tomograms were collected and reconstructed to generate 3D image stacks. To provide a three-dimensional overview of the whole CNS we chose a representative sample and segmented an image stack to label the gross structures, revealing the arrangement of the eleven individual ganglia and their principal nerve tracts (Fig. 1*B*). The resolution of this full-CNS dataset is also sufficient to allow detailed examination of the arrangement of individual cell bodies (Fig. 1*C*). Next, to explore design principles of molluscan CNS organization, we focused on the buccal ganglia, paired spherical structures that house the main feeding circuits, and the subject of intensive characterization over more than six decades (18, 26, 29, 33, 37, 39). Nonetheless, despite this body of work, as in most model systems, the neural tissue is largely unmapped, the total component number is unknown, and broader organizational features remain poorly defined. To address this, we collected high-resolution image stacks and used semiautomated segmentation to label cell bodies (Fig. 1*D*), yielding a complete three-dimensional model of the paired ganglia (Fig. 2 *A*–*E* and Movie S1). Reconstructions show that cell bodies decorate all faces of the ganglia, being excluded only from the entry and exit points where the major nerve bundles pass through the ganglionic core. This detailed annotation allowed us to make an accurate assessment of the total number of neurons contained in the ganglia, yielding a count of 1,099 cells. Such a value is significant because it exceeds a previous estimate (41) by more than three-fold, suggesting that the structural complexity of these ganglia has been significantly underrepresented to date. Within this total population, we observed clear heterogeneity in individual cell soma volumes, with values varying over more than two orders of magnitude (Fig. 2*F*). Notably, the cell count and distribution of somal volumes were almost identical between left and right sides (Fig. 2*G*), confirming that these ganglia have clear bilateral symmetry. On the other hand, this size heterogeneity was not represented evenly in different regions of one ganglion; cell bodies on the dorsal face, for example, were significantly larger, on average, than those on the ventral surface (Fig. 2*H* and *SI Appendix*, Fig. S1).

Most functional investigation of feeding circuitry in *Lymnaea* has focused principally on cells located on the ganglionic surface. As such, the possibility that a significant fraction of neurons might be fully contained inside the ganglia, and therefore largely overlooked in classical electrophysiological interrogation based on superficial recordings, deserves attention. Here, we computed the distance from each somal volume to its nearest point on a mesh describing the ganglionic surface and used this measure to selectively separate the superficial and nonsuperficial populations for further analysis (Fig. 2*I*). This revealed that almost half of all neurons (48%) were, in fact, fully contained inside the ganglionic volume (Fig. 2*I*, *Bottom*, Fig. 2*J*, *Left*, Fig. 2*K* and Movie S2). These nonsuperficial cell somata were, on average, significantly smaller than those decorating the surface, with a narrower distribution of volumes (Fig. 2*J*, *Left*, *Inset*). Furthermore, there was an inverse correlation between somal volume and increasing ganglionic depth; in other words, even among the nonsuperficial neuron population, the smallest cell bodies lie deepest inside the ganglia (Fig. 2*J*, *Right*). We next address the possibility that the largely uncharacterized neurons inside the ganglia can make significant contributions to function. Taken together, SXRT provides a powerful approach to capture micrometer-level detail in multimillimeter scale brains and here reveals previously unreported organizational principles of the molluscan CNS.

### A Major Class of Command-like Feeding Neuron Identified with the SXRT Atlas.

**Fig. 3.**
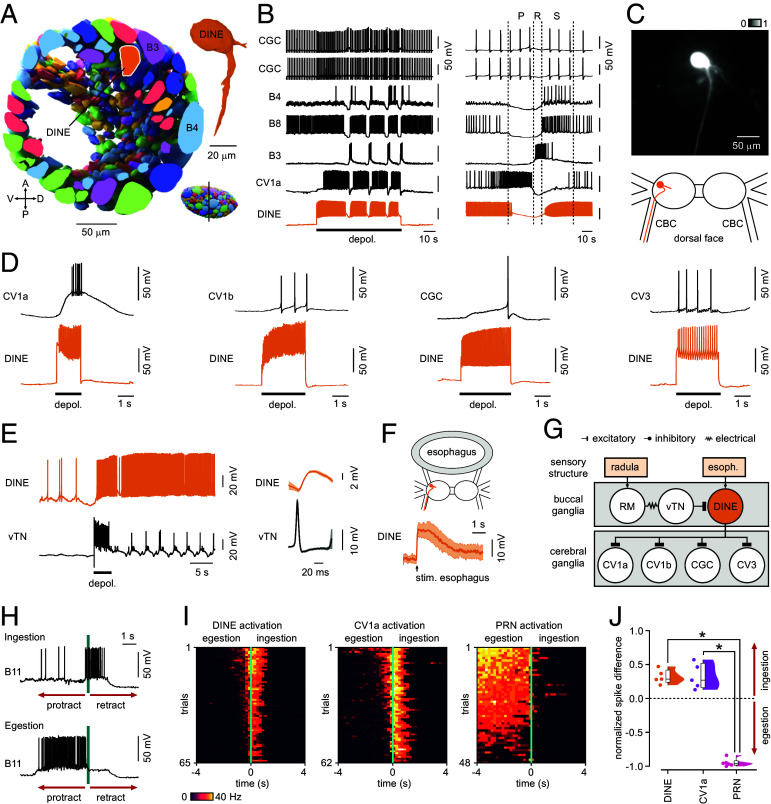
A class of command-like feeding neuron identified with the SXRT atlas. (*A*) Cross-section through reconstructed right buccal ganglion (slice indicated in *Bottom Right*) showing the position of a nonsuperficial neuron (“DINE”) that lies below the landmark superficial motoneuron, B3. The *Top Right* inset shows reconstruction of DINE with its major projection toward cerebro-buccal connective (CBC). A: anterior, P: posterior, D: dorsal, V: ventral. (*B*) (*Left*) Artificial activation of DINE drives fictive feeding cycles indicative of a command-like role. (*Right*) Detail of patterning indicates that DINE is active in the initial protraction phase of the feeding cycle and throughout the swallow phase. P: protraction, R: rasp, S: swallow. (*C*) Alexa Fluor 568 dye-filling confirms SXRT reconstruction revealing a single, long projection down the CBC into the cerebral ganglion. (*D*) Pairwise recordings show that DINE has powerful excitatory connections with command-like interneurons and modulatory cells in the cerebral network (DINE → CV1a, n = 5; DINE → CV1b, n = 4; DINE → CGC, n = 6; DINE → CV3, n = 3). (*E*) (*Left*) The command-like interneuron, vTN, relaying sensory input from the radula (38), strongly activates DINE, revealing 1:1 EPSPs (n = 9). (*Right*) Waveforms showing mean activity profiles ± SEM for n = 10 vTN spikes and DINE responses, aligned to the peak times of the vTN spikes. (*F*) DINE is also strongly excited by stimulation of the esophagus (n = 4), suggesting that this neuron is acting as an integrator of sensory input. Waveform is mean activity profile ± SEM for n = 5 responses. (*G*) Summary schematic illustrating DINE input–output pathways. (*H*) Recordings showing B11, a phase-switching motoneuron whose activity can be used to discriminate between ingestion and egestion cycles, determined as in (22). (*I*) Heatplots of B11 activity for DINE (n = 5 preparations) and CV1 (n = 5 preparations) -activated cycles show that both exclusively promote ingestion responses, while the command-like interneuron, PRN, only drives egestion responses (n = 6 preparations). Trials were sorted according to total activity in feeding cycles. (*J*) Quantification of (*I*) showing normalized B11 spike count differences in ingestion versus egestion for each neuron type activated (one-way ANOVA, *P* < 0.001 [*F*(2, 13) = 197.7]; Tukey’s test: DINE versus PRN, *P* < 0.001; CV1a versus PRN, *P* < 0.001; DINE versus CV1a, *P* > 0.05).

Feeding behavior in *Lymnaea* consists of rhythmic movements of the buccal mass and radula and is controlled by a triphasic central pattern generator (CPG) located in the buccal ganglia (19). Importantly, fictive feeding cycles can be driven in isolated CNS preparations by activating higher-order interneurons, allowing a full interrogation of the contribution that individual neurons make to behavior generation. Our cell atlas uncovers a large population of cells contained inside the ganglionic structure whose involvement in central patterned feeding output is unknown. Here, to investigate this, we functionally targeted a nonsuperficial cell using the positions of surface-level neurons as landmarks (Fig. 3*A*). Intracellular recordings using sharp microelectrodes showed that this neuron was a functionally distinct and uncharacterized cell type with a profound influence on the feeding network, which we have termed “DINE” (Diamond Neuron). Specifically, artificial activation of DINE by depolarizing current injection was sufficient to drive feeding cycles, monitored by the rhythmic bursting of corecorded feeding neurons, for the full stimulus duration, suggesting a command-like role in eliciting and sustaining behavioral expression (Fig. 3*B*). Fluorescent dye-filling confirmed the morphology seen in our SXRT atlas and revealed an unusual projection to the cerebral ganglion which forms part of the ganglionic ring in the main CNS (Fig. 3*C*). Notably, DINE had powerful excitatory connections with command-like interneurons and modulatory cells in the cerebral network but no direct inputs onto neurons in the buccal ganglia where its own cell body is located (Fig. 3*D* and *SI Appendix*, Fig. S2). As such, its ability to drive rhythmic activity in buccal neurons is achieved via its recruitment of the feeding command center located in the cerebral ganglia. Given this atypical arrangement, we hypothesized that DINE may serve to convey input from sensory structures that arrive in the buccal ganglia to the rest of the CNS. Consistent with this, DINE is powerfully excited by radula mechanosensory neurons (RM neurons) through its activation of the command-like interneuron, vTN (ventral Trigger Neuron) (22, 38) (Fig. 3*E*). Likewise, esophageal stimulation also drives strong activation of DINE (Fig. 3 *F* and *G*). Given that the buccal feeding circuitry is known to drive two incompatible behaviors, ingestion or egestion, we next investigated which output pattern is elicited by DINE. Using the phase-switching motoneuron B11 to discriminate between these behaviors (Fig. 3*H*) (22), we show that DINE exclusively drives ingestion feeding cycles (Fig. 3 *I* and *J*), consistent with its excitatory connection with ingestion (CV1a) but not egestion (PRN) interneurons (22). Taken together, we have identified a decision-making element in the circuit which acts as a key relay between sensory input and pattern generation.

### SXRT-Mediated Identification of a Feeding Central Pattern Generator Interneuron.

**Fig. 4.**
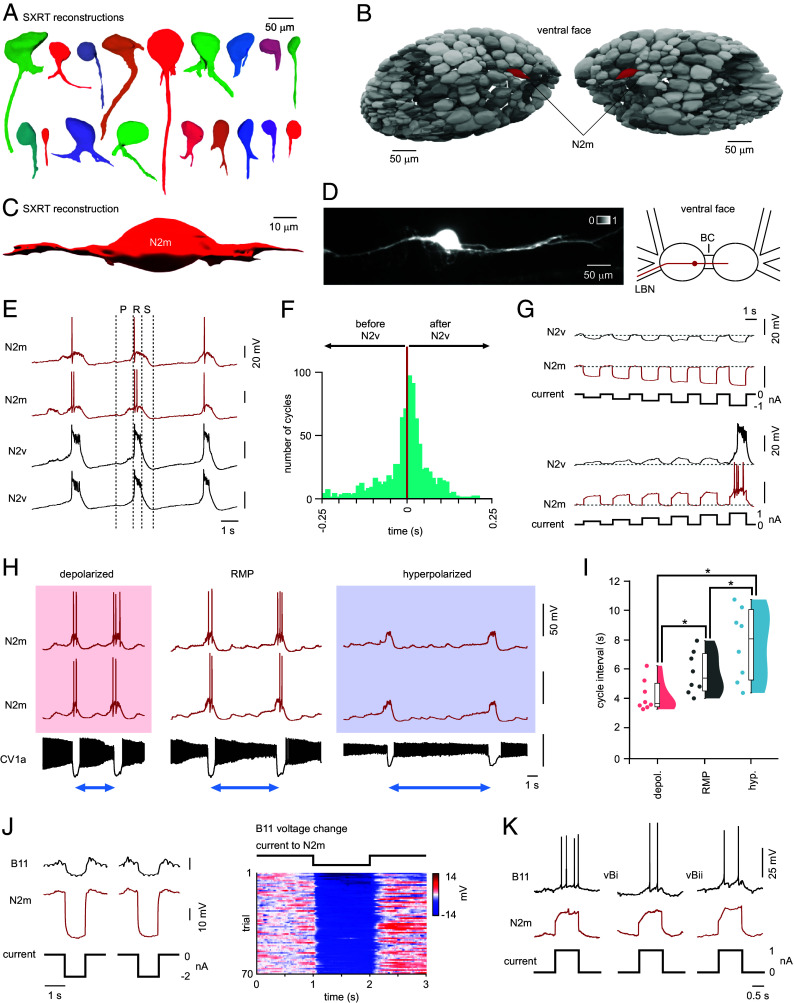
Characterization of a feeding central pattern generator interneuron, N2m. (*A*) Examples of different cell projection morphologies seen in buccal neurons from SXRT reconstructions. (*B*) Positions of N2m, a bilaterally symmetrical pair of neurons, and (*C*) reconstruction revealing an elongated bipolar configuration with bidirectional projections along the medial–lateral axis. (*D*) N2m anatomy based on Alexa Fluor 568 dye-filling showing branches exiting the ganglion through the buccal commissure (BC) and along the lateral buccal nerve (LBN) (n = 7). (*E*) Simultaneous recordings (*Left* and *Right*) showing that N2m is rhythmically active during feeding cycles producing large action potentials during the second, rasp phase. P: protraction, R: rasp, S: swallow. (*F*) Summary of N2m firing activity relative to the other prominent N2 class CPG interneuron, N2v, showing that N2m is active ahead of N2v in around 43% of cases (based on analysis of 760 feeding cycles from n = 4 preparations). (*G*) N2m displays strong electrical coupling with N2v which can trigger a plateau potential in N2v (coupling coefficient: ~12% under subthreshold conditions). (*H*) Manipulation of N2m activity alters cycle length in CV1a-driven feeding rhythms. Depolarization of the *Left* and *Right* neuron pair significantly reduced feeding cycle interval and hyperpolarization significantly increased it. Neurons held at resting membrane potential (RMP) showed intermediate feeding cycle intervals (n = 8). (*I*) Population analysis quantifying feeding cycle interval changes due to depolarization or hyperpolarization of N2m [Friedman test, *P* < 0.001 (χ^2^ (2) = 16), Wilcoxon pairwise: RMP versus depolarized, *P* < 0.05, RMP versus hyperpolarized, *P* < 0.05, depolarized versus hyperpolarized, *P* < 0.05]. (*J* and *K*) Strong electrical coupling from N2m to motoneurons. (*J*) Connection with B11 shows ~23% coupling coefficient (*Left*) (n = 5) and, when hyperpolarized, consistently hyperpolarizes B11 membrane potential in repeated trials, shown in the heatplot (*Right*) (n = 7). Trials were sorted according to the hyperpolarization level recorded in the trial. (*K*) Subthreshold depolarizing current steps in N2m are sufficient to drive action potentials in target motoneurons.

Our cell atlas offers detail on cell body organization and provides insights into features of primary neurites. It reveals that a typical neuron in the buccal ganglia exhibits a unipolar configuration with a single projection from the cell body, often bifurcating further down (Fig. 4*A*). However, deviations from this template are also observed: One notable example revealed when surveying neurons reconstructed in our SXRT reconstruction was a bilaterally symmetrical cell pair lying on the ventral buccal surface with an elongated bipolar configuration and projections extending in opposite directions along the medial–lateral axis (Fig. 4 *B* and *C*). Given this unusual and distinctive arrangement, we set out to functionally characterize this neuron type—guided by our atlas—using intracellular recording approaches. Once located, we confirmed the same anatomical organization using dye-filling, showing that its projections exit the ganglia in one direction through the buccal commissure to the contralateral ganglion and, in the other, along the lateral buccal nerve (Fig. 4*D*). Next, we demonstrated that this neuron plays a key role in feeding central pattern generation. Specifically, during fictive feeding cycles, it produced action potentials during the second (N2, rasp) phase (Fig. 4*E*). This neuron was active before the principal N2-phase CPG interneuron, N2v (42), in approximately half of feeding cycles indicating that it plays a key role in N2 phase initiation (Fig. 4*F*). Moreover, when artificially activated it triggered a plateau potential in N2v, mediated through a strong electrotonic connection (Fig. 4*G*, coupling coefficient: ~12%), and initiated N2 phase inputs across other buccal neurons. Based on this, we hypothesized that manipulation of its activity should alter cycle length in feeding rhythms. Depolarization of the left and right neuron pair during ongoing fictive feeding significantly reduced the time between cycles by bringing on the N2 phase earlier, while hyperpolarizing them significantly increased the cycle interval by delaying N2 onset (Fig. 4 *H* and *I*). Strikingly, this neuron also displayed very strong electrical coupling to motoneurons (B11, ~23% coupling coefficient) which enabled even subthreshold depolarizations to drive spiking activity in coupled motoneurons (Fig. 4 *J* and *K*). This is significant because the membrane potential depolarization experienced by this neuron during feeding rhythms is ~20 mV suggesting that its subthreshold electrotonic recruitment of motoneuron activity likely plays a key role in generating motor activity. Taken together, we propose that this cell is a previously unreported and key member of the feeding central pattern generator, contributing to the second phase of the three-phase rhythm. Based on its medial location in the ganglia, as well as its connectivity, phase of activity, and modulatory influences, we term this neuron N2m.

### A Behavior-Dependent Class of Feeding Motoneuron on the “Dark Side” of the Buccal Ganglia.

**Fig. 5.**
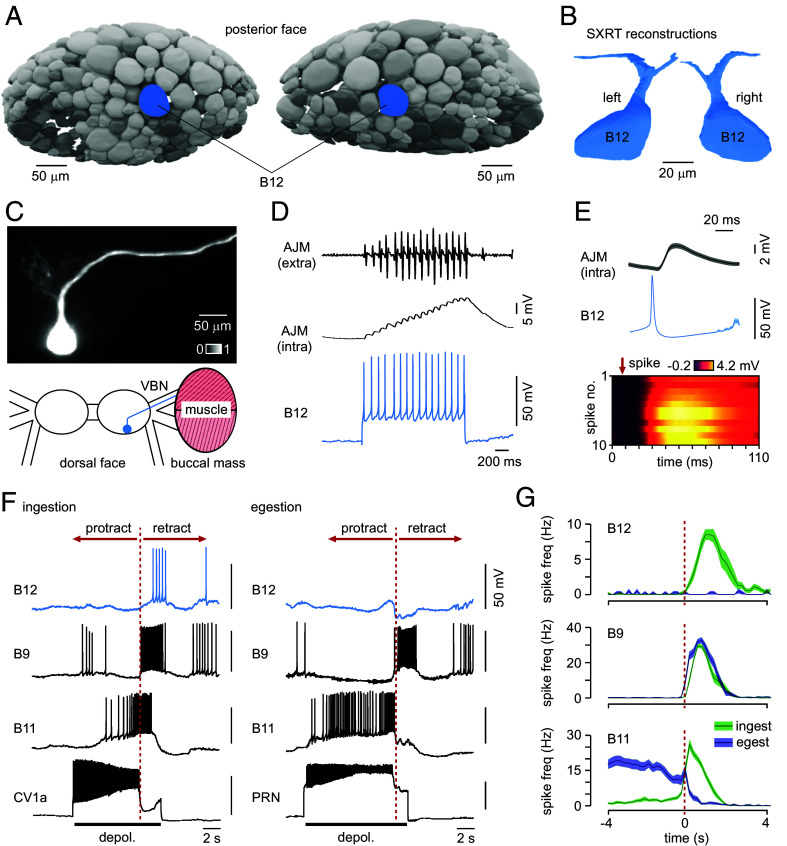
A class of behavior-specific feeding motoneuron, B12. (*A*) The SXRT cell atlas reveals the organization of the uncharacterized posterior face of the buccal ganglia with positioning of a feeding motoneuron class, B12, indicated in blue. (*B*) Reconstructions of B12 in both ganglia show its characteristic morphology with a bifurcating projection architecture. (*C*) Details of B12 anatomy based on Alexa Fluor 568 dye-filling, showing its main projection via the ipsilateral ventral buccal nerve (VBN) to the buccal mass (n = 6). (*D*) Action potentials triggered in B12 cause fast excitatory responses in the major retraction phase muscle (anterior jugalis muscle, AJM) confirming B12 as a motoneuron (n = 5). (*E*) (*Top*). B12 action potentials drive 1:1 excitatory junction potentials in AJM. Waveforms show mean activity profiles ± SEM for n = 11 responses. (*Bottom*) Heatplot shows consistency of excitatory responses evoked in target muscle across trials. (*F*) Recordings showing a behavior-specific role for B12 in feeding-related rhythms. (*Left*) B12 is active in the retraction phase (late rasp and swallow phases) of CV1a-driven ingestion cycles. (*Right*) B12 is completely inactive in PRN-driven egestion cycles. The dashed red line indicates onset of the N2 phase. (*G*) Summary plots quantifying behavior-specific activation of B12 compared to two other motoneuron classes with different response profiles. Waveforms show mean activity profiles ± SEM. B12: n = 7 preps (27 ingestion, 21 egestion cycles), B9: n = 7 preps (29 ingestion, 24 egestion cycles), B11: n = 7 preps (28 ingestion, 28 egestion cycles).

Our cell atlas enables exploration of ganglionic “dark sides” - faces that are normally largely hidden from view owing to the arrangement of nerve bundles that anchor the CNS and obscure these regions. Reconstruction of the posterior face, for example, reveals the organization of one such area. Here, we used this insight to inform development of a twisted CNS preparation that exposes this region for targeted intracellular electrophysiology and revealed a consistently positioned neuron type (Fig. 5 *A* and *B*). Dye-filling demonstrated that this cell had a single projection, leaving the buccal ganglia via the ipsilateral ventral buccal nerve (VBN) toward the buccal mass (Fig. 5*C*). When action potentials were triggered, they caused fast 1:1 excitatory responses in the major retraction phase muscle (anterior jugalis muscle, AJM) indicating that this cell is a motoneuron (Fig. 5 *D* and *E*). Applying the feeding motoneuron nomenclature used in *Lymnaea*, we therefore named it B12. During CV1a-driven ingestion cycles, we found that B12 was active during the retraction phase (late rasp and swallow phases), in agreement with the role of its target muscle (AJM) in retracting the buccal mass and odontophore/radula during feeding (Fig. 5*F*, *Left*, Fig. 5*G*). However, during PRN-driven egestion cycles, B12 showed no activity, instead receiving a large inhibitory input throughout the retraction phase (Fig. 5*F*, *Right*, Fig. 5*G*). B12 is therefore an example of a behavior-specific motoneuron that is only recruited into ingestion cycles. As such, while some core features of the feeding-related circuit are shared across both behaviors—for example, activity in neurons B9, N2v, N1M (22)—behavior-specific recruitment of a subset of neuron types [e.g., B12, PRN, CV1a (22, 39)] and reconfiguration of others [e.g., phase-switching of B11 activity (22)] differentiate them (Fig. 5*G*). A behavioral outcome of the differential recruitment of B12 that we observe here is that ingestion is likely to be associated with stronger/successful swallowing during ingestion and reduced radula retraction during egestion, thus preventing the animal from swallowing potentially harmful objects.

### Atlas Building in the *Lymnaea* CNS.

**Fig. 6.**
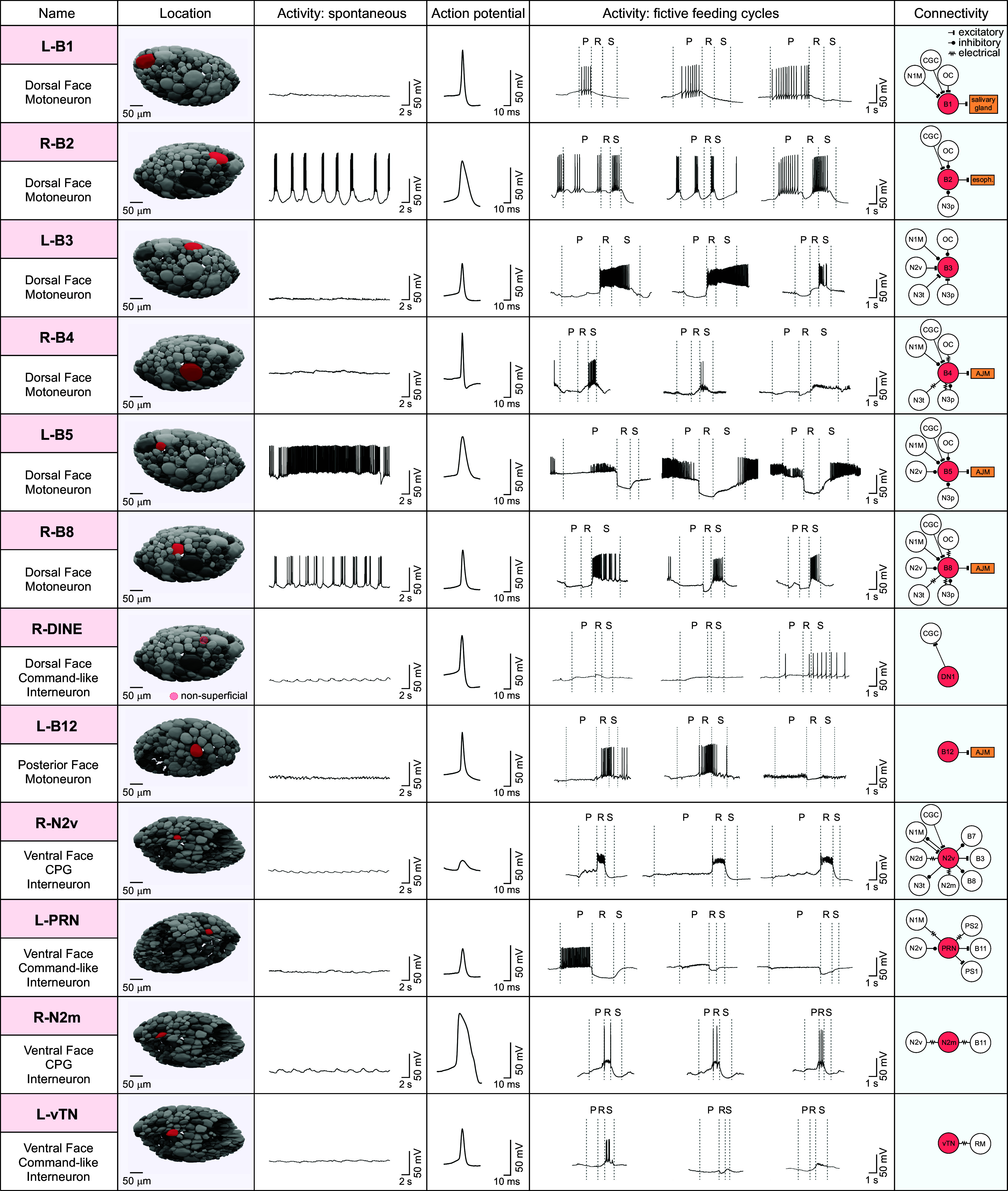
An SXRT-based atlas of key cell types in the buccal ganglia. Examples of a subset of prominent neuron types with detail on their position in the ganglionic structures based on SXRT reconstruction, their electrophysiological properties, and their known connectivity. A comprehensive summary of known buccal neuron types is provided in *SI Appendix*, Figs. S3–S5. For electrophysiological recordings (columns 3 to 5), typical examples show spontaneous activity in healthy neurons, their average action potential waveforms (n ≥ 3), and three examples of activity during fictive feeding cycles, illustrating diversity of response profiles. The right column summarizes their known connectivity to key feeding interneurons, central pattern–generating interneurons N1M, N2v, N3p, and N3t, and modulatory interneurons CGC and OC (30, 33, 42–47).

*Lymnaea* offers rich opportunities for atlas-building using SXRT thanks to the identified nature of its neurons. For many cell types, detail contained in our reconstructions relating to the position, size, and anatomical projection properties is sufficient to confirm a match with specific neurons recorded and labeled in electrophysiological studies, allowing this information to be aggregated and organized in a functional atlas. We showcase the generation of such a resource here by mapping the locations of 36 of the principal feeding-circuit cell types, including motoneurons, CPG neurons, and modulatory cells, alongside multiple recordings of each type during spontaneous and evoked activity, giving a detailed summary of their main functional properties (Fig. 6 and *SI Appendix*, Figs. S3–S6). Some of these cells have been previously identified (19) and are shown here in the context of their detailed spatial position in the ganglia. Others, including DINE, N2m, B12, vBC1, and vBC2 (*SI Appendix*, Fig. S7), are cell types that we have characterized in the present study. Such a database is fully scalable allowing additional information and cell types to be added and will substantially assist the community engaged in circuit studies, reducing the uncertainty about how to find and confirm recordings of specific cell types. This contrasts with the present ad hoc strategies to identify neurons, where researchers typically refer to cartoon representations of neuron positions with little or no clear contextual information available. Our approach here provides the pipeline for generating a whole CNS map for detailed functional characterization of all behavior-generating networks. Importantly, given that our imaging method is noninvasive and directly compatible with processing steps needed for electron microscopy, it provides a valuable means to capture gross neuronal morphology that could then be used to guide follow-up ultrastructural interrogation in the same sample.

## Discussion

We have established a generalizable methodological pipeline based on SXRT that enables rapid CNS atlas-building in multimillimeter-scale brains, structures for which state-of-the-art mapping techniques using volume electron microscopy approaches still remain very challenging, labor intensive, and largely exceed the requirements for capturing the organization of circuit components for functional interrogation. Given the surfeit of model organisms in neuroscience research, we suggest that SXRT imaging has significant value in enabling the generation and sharing of whole-CNS models of a variety of species. This method has particular value for systems that have “identified” neurons—those which occupy essentially the same location within the CNS, and with the same morphological and biophysical features in different individuals (6, 48–60)—a characteristic typical of many simpler model organisms used for circuit investigations. In this way, cells of interest defined by their positional and morphological characteristics can be easily targeted in recording experiments allowing a functional atlas, both for sharing and organizing data across research groups, to be readily compiled.

We showcase this general approach for rapid atlas-building using *Lymnaea*, an exceptionally accessible and extensively investigated molluscan system for interrogating fundamental circuit mechanisms associated with decision-making, pattern generation, and learning and memory (17, 18, 20–22, 27, 28, 30, 32–34, 37–39, 61–63). Focusing on the reconstruction of the paired buccal ganglia that house the main feeding circuitry—the best-characterized network in this organism—we provide a comprehensive estimate of the total neuron number (~1,100), revising the cell count upward approximately threefold from a previous estimate (41), and suggesting that the complexity of these structures has been significantly underappreciated to date. We also reveal insights into the organizational principles linking cell body volumes to their ganglionic positions, demonstrating significant differences in cell sizes across different ganglionic faces and an inverse relationship between increasing depth from the ganglionic surface and somal volume. We suggest that there may be potential operational advantages in such a design for a small nervous system. With the largest cell bodies present near the ganglionic surface, all neurites can project to the nerve tract in the core without facing the largest obstacles, avoiding the need for detours and thus minimizing wiring length and brain volume (64). Moreover, with the largest cell bodies residing at the surface, the center of the ganglia can thus be reserved for high-density neural wiring. Such an arrangement increases the number of possible interactions between neurites—via chemical and electrical synapses—within a given distance range, providing the substrate for more complex logical operations at lower energy cost (65). Applied to other neural systems our approach may reveal related, fundamental design rules for brain architectures or hint at their emergence over evolutionary timescales (66).

We provide several demonstrations of the value of our cell atlas for rapid functional insights. Specifically, we significantly update our understanding of the feeding circuit by identifying and characterizing three cell types: a command-like modulatory neuron, a principal central pattern–generating interneuron, and a unique behavior-specific motorneuron, each revealed through structural insights gained from our 3D map. Characterization of the command-like neuron emerged from our observation that a significant fraction of neurons (~50%) lie fully contained within the ganglionic volume and thus, to date, lack functional designations. Here, using intracellular recording methods and guided by the detail in our map, we identified and classified DINE, a neuron type with striking functional properties that links sensory input arriving from the mouth and esophagus to the generation of full CPG-driven fictive feeding. We propose that this neuron plays a pivotal role in coupling the detection of food reported by mechanoreceptors when animals carry out sampling feeding events to the recruitment of the ingestion circuitry required to drive a full feeding rhythm. In view of its functional role, “DINE” is a highly appropriate name for this cell type, forming a major part of the circuit that enables feeding expression when suitable material is ingested during food-searching.

The SXRT images also provide sufficient resolution to visualize broad anatomical features of individual cells. Scrutiny of these led us to identify an important interneuron type, N2m, revealed by its unusual bipolar projection, which we determined to be a significant member of the core CPG circuit that drives feeding behavior. Notably, this neuron type has dual output properties: Both digital, as action potentials, and analog, via its electrotonic connections. In particular, the exceptionally strong electrical coupling between this neuron and target motoneurons allows its subthreshold depolarization alone to drive full motoneuron spiking activity. Such a mechanism, in which spiking is not necessary to achieve profound influences on motor patterning, has not been previously characterized in *Lymnaea*, but extends earlier work in this system suggesting that CPG interneurons and motoneurons are closely coupled to allow tight output synchronization (30). We note that in the marine mollusk *Aplysia*, a neuron with a similar bipolar morphology, B21, has a profound effect on the feeding circuitry in part due to subthreshold changes in somatic membrane potential (67) suggesting that this anatomical feature could be a target for identifying influential neurons in molluscan preparations.

Our map also provides a detailed view of the neuronal organization of ganglionic regions that have so far remained largely uncharted thanks to the way that nerve bundles exit the ganglia and thus typically hide some faces from view—we refer to these as ganglionic “dark sides.” Our atlas allows us to examine the detailed organization of neurons in these regions and thus inform functional targeting, leading to the characterization of a type of feeding motoneuron, B12. Notably, this is an example of a behavior-specific motoneuron in the *Lymnaea* feeding CPG, in this case recruited into ingestion cycles but not egestion cycles. A likely behavioral consequence of this differential activation is that swallowing actions are enhanced in ingestion but suppressed in egestion, preventing the animal from ingesting potentially harmful objects.

SXRT is emerging as a powerful tool for mapping components of mammalian networks (15, 16, 68) but has not been exploited widely for interrogating the brains of simple model organisms. We anticipate that the approach we outline here will readily generalize to various model systems with comparable brain sizes (e.g., other mollusks, crustacea, annelids). For other animal classes, such as many insects, where neurons are often too small for SXRT to reliably resolve detail, it can provide a rapid means to gain overview maps, for example, of neuropil organization, fiber tracts, soma clusters, and other structural features. Since the datasets that it yields are relatively small, especially when compared to the nanoscale maps that emerge from the extraordinary but labor-intensive electron microscopy-based connectomics approaches, the generation of an accurate overview atlas is rapid and easy to interrogate, aiding comparative studies and detailed functional characterization. For example, the convenience of this approach would enable rapid investigation of interindividual variability within a species. Sharing maps across the community can also benefit from the development of open-source GPU-accelerated image-sharing platforms (e.g., Webknossos) (69) which provide powerful tools to engage with raw image stacks, annotate structures, and quickly build informative 3D reconstructions. Taken together, we anticipate that our methodological pipeline will be invaluable for comparative neurology studies, revealing principles of CNS design in different animal phyla and the way that these are shaped by evolutionary processes (70).

## Materials and Methods

### Animal Maintenance.

*L. stagnalis* were maintained on a 12:12 h light–dark regime in large holding tanks containing Cu^2+^-free water at 20 °C. They were fed a diet of vegetable-based fish food (Tetra-Phyll; TETRA Werke, Melle, Germany) and lettuce. All experiments used adult (3 to 4 mo old) animals. *Lymnaea* is a lower invertebrate (molluscan) organism that does not fall under The Animals (Scientific Procedures) Act (ASPA) 1986 (UK) and so ethical approval or guidance was not required for these experiments. Nonetheless, experiments underwent an institutional Non-ASPA ethical review at the University of Sussex (ARG-32-KS) that covers this work.

### SXRT Imaging and Reconstruction.

For X-ray imaging experiments, the CNS was isolated in saline with composition (in water) as follows: 50 mM NaCl, 1.6 mM KCl, 2 mM MgCl_2_, 3.5 mM CaCl_2_, and 10 mM HEPES buffer. After dissection, samples were fixed overnight in 2% paraformaldehyde and 1.5% glutaraldehyde in 0.1 M phosphate buffer. To prepare for synchrotron X-ray tomography imaging, a modified version of the NCMIR protocol (https://ncmir.ucsd.edu/sbem-protocol), designed to enhance the membrane contrast, was used. Details are provided in Supporting Information. Resin-embedded samples were transferred to the Diamond Light Source National Facility and tomographic measurements were performed at the I13-2 beamline, using the so-called “pink X-ray beam.” The energy bandwidth of the radiation from the undulator source was limited by the combination of X-ray filters (1.34 mm carbon, 3.2 mm aluminum) for lower energies and a (horizontally) deflecting Pt mirror for high energies. With the given settings and an undulator gap of 5 mm, the average weighted mean photon energy was 27 keV. For each tomography dataset, 3001 projections were recorded at equidistant viewing angles (0.06° steps) over 180° of sample rotation. Images were acquired using 2× and 10× objectives with exposure time 0.47 s per image for 10× objective (total scan time ~20 mins) and 0.065 s for 2× objective (total scan time ~3 mins). Tomographic reconstructions were obtained using Savu software developed at Diamond Light Source; cubic voxels were approximately 0.325 µm (10× objective) and 1.625 µm (2× objective). Image stacks were imported into WEBKNOSSOS (webknossos.org)(69), an open-source tool for annotating 3D image stacks and for generating 3D meshes. Cells were identified by their broadly spherical somal structures (>8 µm), visible nuclei, and projection anatomy and then digitally annotated. Boundaries of ganglionic structures were defined by their superficial cell bodies. The neuropil, contained inside the ganglionic volume and housing the principal neuronal wiring, was defined by its fine wiring structure and the absence of cell bodies. Labeling was achieved by manually marking cell boundaries and then utilizing a segment interpolation feature to semiautomate the process across sections. For annotations used for volume measurements, all fine neuritic processes (<~2.5 µm) were excluded and the cell body boundaries were defined using a threshold indicated by segment interpolation in sections where no nerve projection was present. To confirm that all visible cell-like structures were neuronal, we carried out an independent electrophysiological screen of cells on the dorsal and ventral surface (diameter range: ~10 to 80 µm). Of 144 cells sampled using intracellular recording approaches, 100% were excitable and able to generate robust action potentials (*SI Appendix*, Fig. S8). Cell meshes were exported to Blender 4.1 (blender.org) to generate renders and to Matlab (mathworks.com) or Mathematica (wolfram.com/mathematica) to enable the quantification of neuronal volumes and organizational relationships. To establish how neuronal properties vary at different depths in the ganglionic volume, the nearest distance from each point on the cell mesh to each point on a smoothed ganglionic surface mesh was calculated using a nearest-neighbor search. Distances of <8.125 µm from the surface were used as the threshold to define the superficial neuron population.

### Functional Characterization Approaches.

Based on previously described procedures (22, 39) we carried out in vitro experiments using either isolated CNS or buccal mass-CNS preparations. The isolated CNS comprised eleven ganglia attached to a small region of the esophagus via the dorsal buccal nerves (DBN). The buccal mass-CNS preparation was used to corecord from muscles (intracellularly and extracellularly) and motoneurons (intracellularly). The CNS was kept attached to the buccal mass via the lateral and ventral buccal nerves. Preparations were perfused with normal saline containing 50 mM NaCl, 1.6 mM KCl, 2 mM MgCl_2_, 3.5 mM CaCl_2_, and 10 mM HEPES buffer in water. Functional assessments of neurons relied on intracellular recordings carried out at room temperature (21 °C) using sharp electrodes (10 to 40 MΩ, filled with 3 M KAc and 0.5 mM KCl). Signals were collected using Axoclamp 2B (Axon Instruments, Molecular Devices) and NL102 (Digitimer Ltd.) amplifiers. Data were acquired at 2 kHz using a micro 1401 Mk II interface and traces were viewed and analyzed using Spike2 software (Cambridge Electronic Design, Cambridge, UK). Muscles were recorded using a sharp electrode or with a glass suction electrode. Signals were amplified using a NL104 (5,000 gain) (Digitimer Ltd.), notch (50 Hz) filtered using NL125/126 filters (Digitimer Ltd.), and then digitized at a sampling rate of 5 kHz using a Micro1401 mK II interface (Cambridge Electronic Design, Cambridge, UK). To test electrical coupling coefficients, each cell was impaled with two electrodes, one for current injection and one for independently measuring voltage changes. Criteria for neuron and muscle recordings, determination of feeding cycles, and cell-filling approaches are provided in Supporting Information.

### Data Analysis.

Data were analyzed using PAST 4.7 (71) and expressed as histograms, scatter plots, or raincloud plots. For raincloud plots, individual data points are dots, and the shaded region (cloud) indicates the overall shape of the distribution extending from minimum to maximum values. Internal boxplots show median (black line) and interquartile range (first and third quartile). Normality was tested using the Shapiro–Wilk test. Two-group statistical comparisons were performed using unpaired two-tailed *t* test statistics or a Mann–Whitney test for nonparametric data. Data with more than two groups were first analyzed using a one-way ANOVA or a Friedman test followed by a Tukey’s post hoc test or Wilcoxon pairwise test with Bonferroni corrected *P* values. Correlation analysis used Spearman’s rank. Significance level was set at *P* < 0.05.

## Supplementary Material

Appendix 01 (PDF)

Movie S1. **3D reconstruction of *Lymnaea* buccal ganglia**. Reconstruction from 10x image stacks of the paired (left and right) buccal ganglia with cell bodies labelled using a randomly-assigned color palette.

Movie S2. **Lateral-to-medial travel through a 3D-reconstructed *Lymnaea* buccal ganglion**. Reconstruction showing the superficial (blue) and internalized (orange) neuron populations in the right buccal ganglion along the lateral to medial axis.

## Data Availability

Excel files containing raw data and an atlas of neuron classes have been deposited in Figshare (https://doi.org/10.25377/sussex.28280456 and https://doi.org/10.25377/sussex.28333793) (72, 73). The synchrotron image stack is available at https://wklink.org/2643 (74). All other data are included in the manuscript and/or supporting information.
