# Supplementary material for: Functional mapping of the molluscan brain guided by synchrotron X-ray tomography
Source: Proc Natl Acad Sci U S A. 2025 Feb 27;122(9):e2422706122. doi: 10.1073/pnas.2422706122 (PMC11892647; doi:10.1073/pnas.2422706122)
Supplement: Supplementary file 1 — Appendix 01 (PDF) [file pnas.2422706122.sapp.pdf]

PNAS

Supporting Information for

Functional mapping of the molluscan brain guided by synchrotron X-ray tomography

Michael Crossley[1*], Anna Simon[2], Shashidhara Marathe[3], Christoph Rau[3], Arnd Roth[2], Vincenzo Marra[1], Kevin Staras[1#]

[#]Corresponding Author and Lead Contact: k.staras@sussex.ac.uk

[*]Corresponding Author: m.crossley@sussex.ac.uk

This PDF file includes:

Supporting Materials and Methods
Figures S1 to S8
Legends for Movies S1 to S2
SI References

Other supporting materials for this manuscript include the following:

Movies S1 to S2

Supporting Materials and Methods

Sample preparation for synchrotron X-ray tomography imaging. Post-fixed samples were washed in cold 0.15 M sodium cacodylate buffer with 3 mM $CaCl_2$ before further fixation at 4°C for 1 hour using 1.5% (w/v) potassium ferrocyanide and 1% (v/v) osmium tetroxide in 0.15 M sodium cacodylate buffer with 3 mM $CaCl_2$. After frequent short washes in double distilled water (ddH_2O) samples were treated with 1% (w/v) thiocarbohydrazide in ddH_2O filtered through a 0.22 µm Millipore syringe filter at room temperature for 15 minutes. Subsequent washes with ddH_2O were followed by further osmication (2% osmium tetroxide in water) for 30 minutes. Samples were washed in ddH_2O, then placed in cold 1% (w/v) aqueous uranyl acetate for 30 minutes followed by ddH_2O rinses. Samples were subsequently dehydrated in an ascending scale of cold ethanol, followed by 100% acetone and infiltrated in 1:3, 1:1, 3:1 Durcupan epoxy resin:acetone (2 hours each). Tissues were kept in 100% Durcupan (11.4 g component A, 10 g component B, 0.3 g component D, 100 µl component C) at room temperature overnight, then placed into fresh 100% Durcupan for 2 hours the following day. For fixation and processing the samples remained pinned on their dissection platform and pins were only removed before the 100% resin infiltration step. Finally, samples were capsule-embedded in freshly made 100% Durcupan resin and polymerized at 60 °C for 48 hours.

Electrophysiological neuron and muscle recordings. The ingestion command-like interneuron, CV1a, is located in the cerebral ganglia and was identified by its characteristic location, electrical properties and ability to drive ingestion fictive feeding cycles when artificially depolarized to fire action potentials (1, 2). The egestion command-like interneuron, PRN, is located on the ventral surface of the buccal ganglia and was identified by its monosynaptic excitatory connection with the B11 motoneuron and its ability to drive egestion fictive feeding cycles when artificially depolarized to trigger action potentials (2). The CPG interneuron, N2v, is located on the ventral surface of the buccal ganglia and was identified by its plateau potential during the retraction phase of fictive feeding cycles (3). Artificial activation of an N2v causes retraction phase activity on many buccal motoneurons and interneurons (4). The phase switching motoneuron B11 is located on the ventral surface of the buccal ganglia and was identified based on its location and synaptic inputs from PRN, and its ability to switch its activity pattern during ingestion and egestion cycles (2, 5). vTN was identified by its location on the ventral surface of the buccal ganglia, its white color, and its response to tactile stimulation of the radula (6). RM is a sensory neuron located on the ventral surface of the buccal ganglia and was identified by its strong connection with vTN and response to tactile stimulation of the radula (2). The CGCs are large serotonergic neurons located in the cerebral ganglia. They were identified by their size, location and synaptic connections with buccal neurons (7). CV1b interneurons were identified by their size, color, and spike shape and their ability to weakly drive fictive feeding cycles (1). The motoneurons B1-B8 and CV3 were identified by their size, location and firing patterns during fictive feeding cycles (8–10). The anterior jugalis muscle (AJM) is a thick

muscle that is involved in the retraction of the buccal mass and radula (2, 10, 11). To test the response of the newly-characterized cell, DINE (see Results), to esophageal stimulation, we stimulated the anterior region of the esophagus using a glass suction electrode (biphasic pulse 10 V, 0.5 ms duration).

Analysis and classification of fictive feeding cycles as ingestion or egestion. Activity of the motoneuron B11 was used to determine whether cycles driven by DINE were ingestive or egestive. B11 activity was measured 4 s before and 4 s after the onset of the retraction phase of driven cycles as in (5). The number of B11 spikes after the onset of the retraction phase was subtracted from the number of B11 spikes before the onset of the retraction phase and then divided by the total number of spikes in the combined 8-s period to obtain a normalized difference score. A positive score value represents more activity occurring before retraction phase onset and therefore is classified as an egestion cycle whereas a negative score represents more activity after retraction phase onset and is therefore classified as an ingestion cycle.

Iontophoretic dye filling of neurons. Following procedures previously described (5), we filled target neurons with fluorescent dye (Alexa Fluor 568, Molecular Probes) using a microelectrode. Neurons were filled iontophoretically using a pulse generator to apply regular interval negative square current pulses. Images of dye-fills used an upright Zeiss Axiophot microscope (x10 dipping objective), excitation: 546/12 nm, dichroic: 580 nm, emission: 590 nm longpass, attached to an Andor Ixon electron multiplying charge-coupled device camera controlled by Micromanager software.

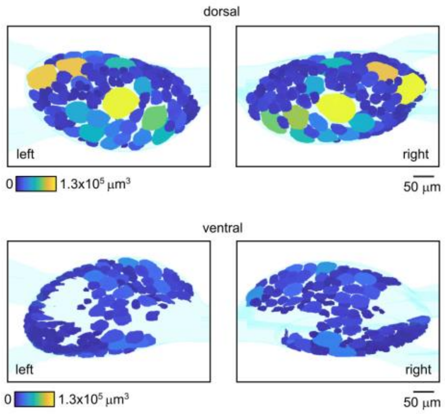

Fig. S1. Variations in cell volumes in buccal ganglia. 3D reconstructions of buccal neurons using color to code for cell body volume reveals consistency across left and right ganglia but significant differences between their dorsal and ventral surfaces (see also Fig. 2h).

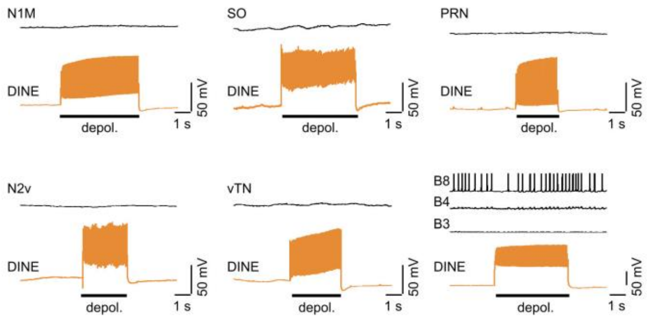

Fig. S2. DINE does not have direct connections to buccal feeding circuitry. Traces show the lack of direct connectivity of DINE onto central pattern generator interneurons (N1M, N2v), command-like interneurons (SO, PRN, vTN) and motoneurons (B3, B4, B8) in the buccal ganglion where its own cell body is located.

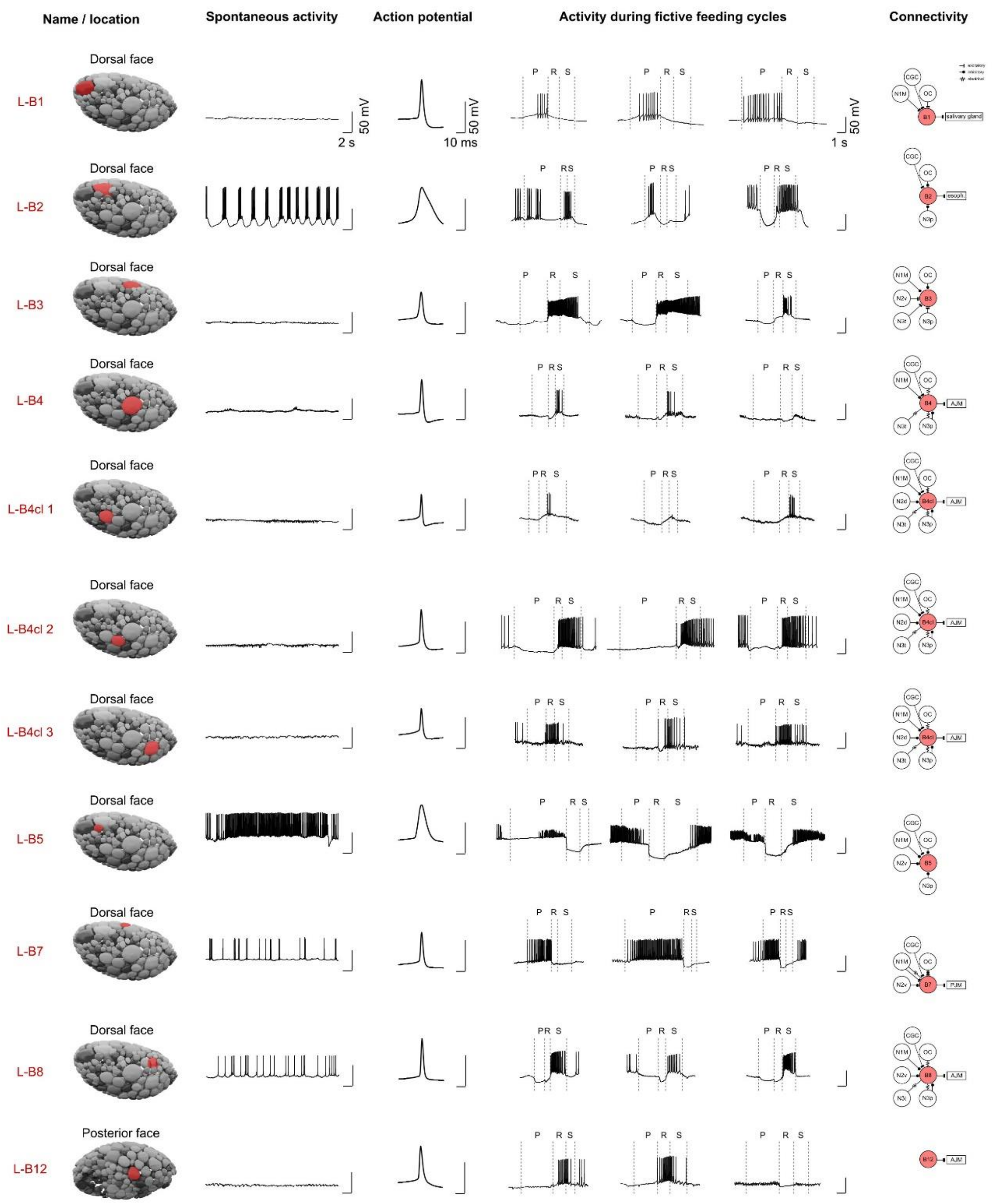

Fig. S3. An SXRT-based atlas of key feeding motoneurons in the left buccal ganglia (dorsal surface). Table shows their positioning on the ganglionic surface and functional profiles based on intracellular recordings. The latter includes typical examples of their spontaneous activity, their average action potential waveform (n >= 3), and three examples of their activity during fictive feeding cycles, illustrating variation in response profiles observed. The right panel summarizes their known connectivity to key feeding interneurons, the central pattern-generating interneurons, N1M, N2v, N3p and N3t, and the modulatory interneurons, CGC and OC (4, 10–16).

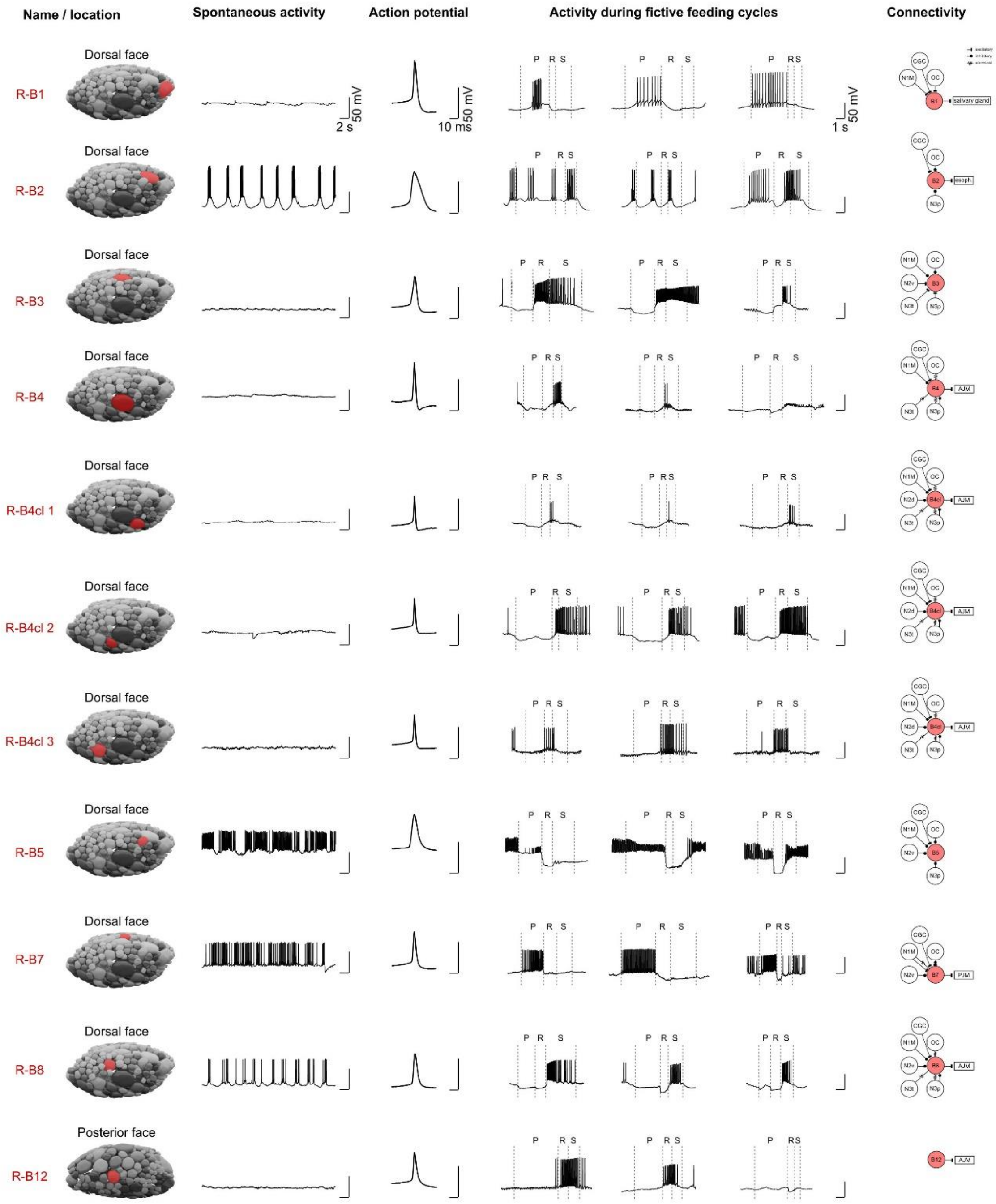

Fig. S4. An SXRT-based atlas of key feeding motoneurons in the right buccal ganglia (dorsal surface). Table shows their positioning on the ganglionic surface and functional profiles based on intracellular recordings. The latter includes typical examples of their spontaneous activity, their average action potential waveform (n >= 3), and three examples of their activity during fictive feeding cycles, illustrating variation in response profiles observed. The right panel summarizes their known connectivity to key feeding interneurons, the central pattern-generating interneurons, N1M, N2v, N3p and N3t, and the modulatory interneurons, CGC and OC (4, 10–16).

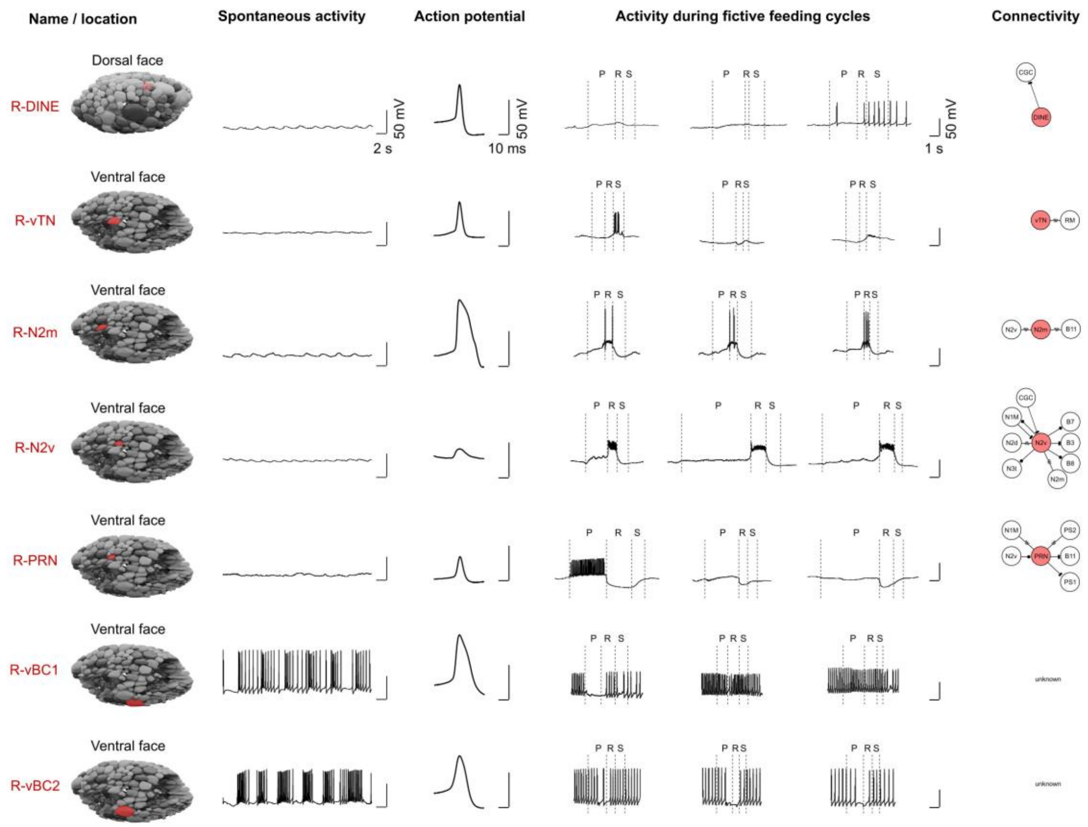

Fig. S5. An SXRT-based atlas of key feeding modulatory and central pattern generator neurons in the left buccal ganglia (ventral surface). Table shows their positioning on the ganglionic surface and functional profiles based on intracellular recordings. The latter includes typical examples of their spontaneous activity, their average action potential waveform (n >= 3), and three examples of their activity during fictive feeding cycles, illustrating variation in response profiles observed. The right panel summarizes their known connectivity to key feeding interneurons, the central pattern-generating interneurons, N1M, N2v, N3p and N3t, and the modulatory interneurons, CGC and OC (4, 10–16).

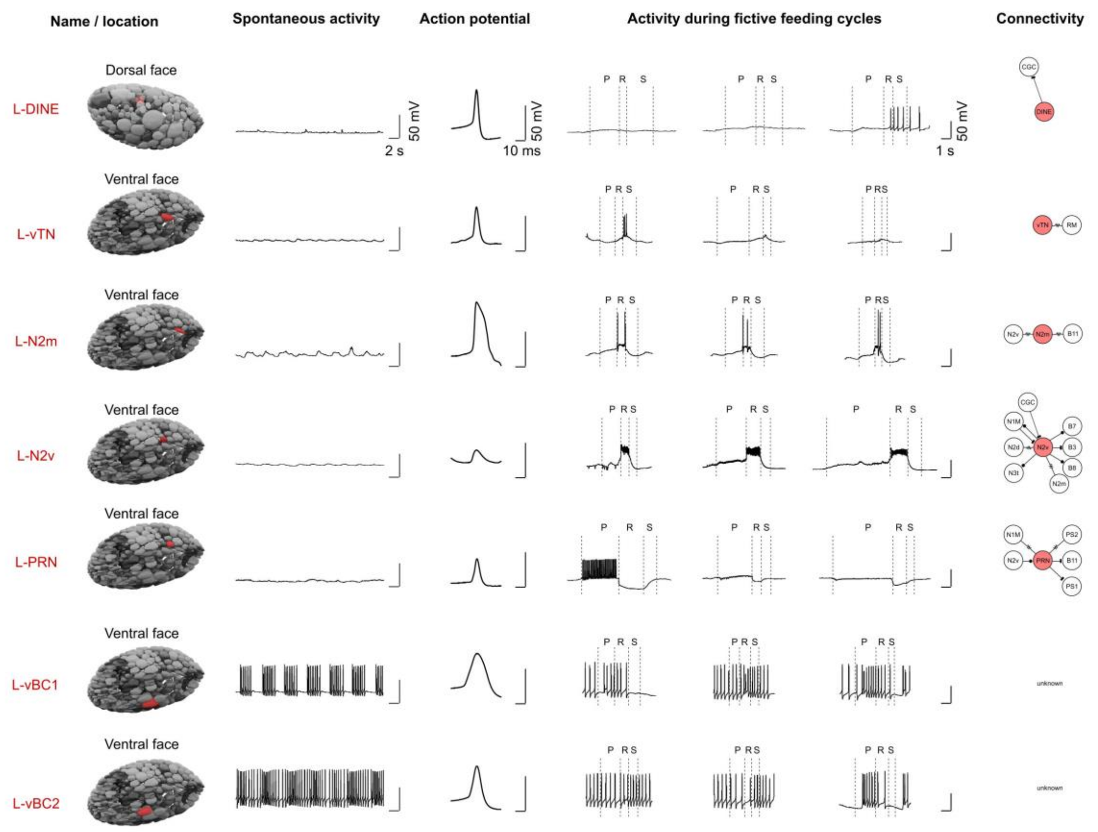

Fig. S6. An SXRT-based atlas of key feeding modulatory and central pattern generator neurons in the right buccal ganglia (ventral surface). Table shows their positioning on the ganglionic surface and functional profiles based on intracellular recordings. The latter includes typical examples of their spontaneous activity, their average action potential waveform (n >= 3), and three examples of their activity during fictive feeding cycles, illustrating variation in response profiles observed. The right panel summarizes their known connectivity to key feeding interneurons, the central pattern-generating interneurons, N1M, N2v, N3p and N3t, and the modulatory interneurons, CGC and OC (4, 10–16).

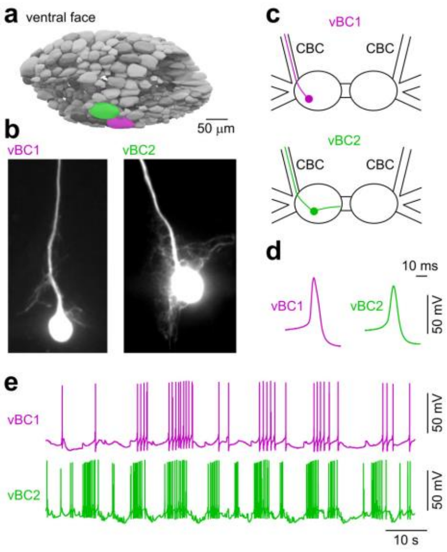

Fig. S7. Initial characterization of landmark neurons on the ventral buccal surface. (a) vBC1 and vBC2 (ventral buccal-cerebral 1 and 2) are morphologically distinct cell-types revealed by our atlas. **(b,c)** Alexa Fluor 568 dye-filling reveals that, like DINE, they have projections down the cerebro-buccal connective (CBC) into the cerebral ganglion. **(d,e)** Targeted intracellular recordings reveal their action potential waveforms **(d)** and spontaneous firing patterns **(e)**.

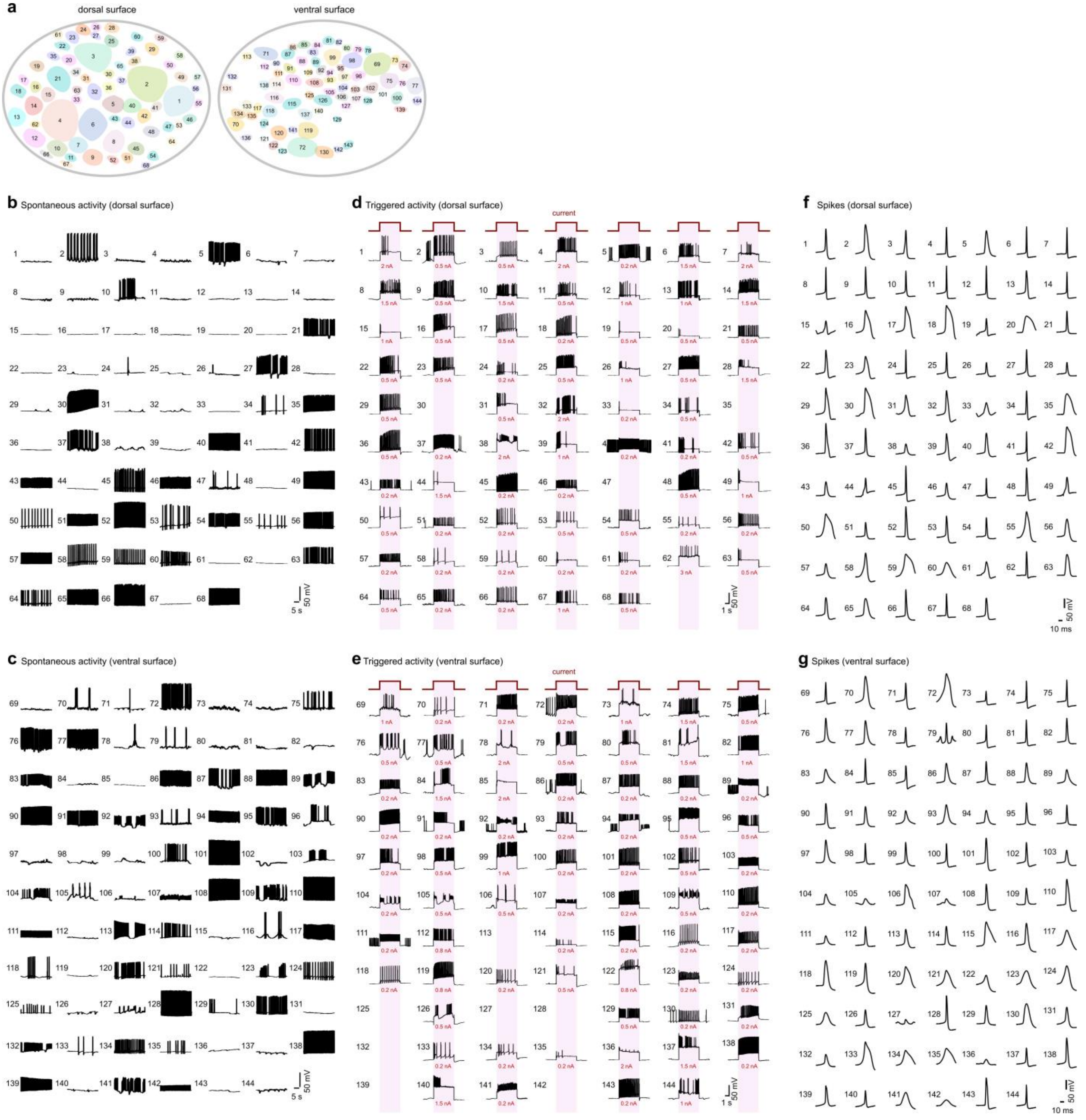

Fig. S8. Electrophysiological confirmation that visible cell-like structures on buccal ganglionic surfaces are neuronal. (a) Using targeted intracellular recording approaches, we carried out an extensive sampling of the visible cell-like structures on the dorsal and ventral ganglionic surfaces to confirm their biophysical properties. For each cell, we recorded spontaneous activity **(b,c)**, evoked activity **(d,e)** and action potential waveform **(f,g)**. 144/144 attempted recordings were successful and in all cases revealed that cells were electrically excitable and able to generate robust action potentials.

Legends for Movies

Movie 1 (separate file). 3D reconstruction of *Lymnaea* buccal ganglia. Reconstruction from 10x image stacks of the paired (left and right) buccal ganglia with cell bodies labelled using a randomly-assigned color palette.

Movie 2 (separate file). Lateral-to-medial travel through a 3D-reconstructed *Lymnaea* buccal ganglion. Reconstruction showing the superficial (blue) and internalized (orange) neuron populations in the right buccal ganglion along the lateral to medial axis.

SI References

1. C. R. McCrohan, M. A. Kyriakides, Cerebral Interneurones Controlling Feeding Motor Output In The Snail Lymnaea Stagnalis. *Journal of Experimental Biology* **147**, 361–374 (1989).
2. M. Crossley, K. Staras, G. Kemenes, A central control circuit for encoding perceived food value. *Sci Adv* **4**, eaau9180 (2018).
3. M. J. Brierley, M. S. Yeoman, P. R. Benjamin, Glutamatergic N2v cells are central pattern generator interneurons of the lymnaea feeding system: new model for rhythm generation. *J Neurophysiol* **78**, 3396–3407 (1997).
4. M. J. Brierley, K. Staras, P. R. Benjamin, Behavioral function of glutamatergic interneurons in the feeding system of Lymnaea: plateauing properties and synaptic connections with motor neurons. *J Neurophysiol* **78**, 3386–3395 (1997).
5. M. Crossley, P. R. Benjamin, G. Kemenes, K. Staras, I. Kemenes, A circuit mechanism linking past and future learning through shifts in perception. *Science Advances* **9**, eadd3403 (2023).
6. M. Crossley, K. Staras, G. Kemenes, A two-neuron system for adaptive goal-directed decision-making in Lymnaea. *Nat Commun* **7**, 11793 (2016).
7. M. S. Yeoman, M. J. Brierley, P. R. Benjamin, Central pattern generator interneurons are targets for the modulatory serotonergic cerebral giant cells in the feeding system of Lymnaea. *J Neurophysiol* **75**, 11–25 (1996).
8. P. R. Benjamin, R. M. Rose, Central generation of bursting in the feeding system of the snail, Lymnaea stagnalis. *J Exp Biol* **80**, 93–118 (1979).
9. C. R. Mccrohan, Properties of Ventral Cerebral Neurones Involved in the Feeding System of the Snail, Lymnaea Stagnalis. *Journal of Experimental Biology* **108**, 257–272 (1984).
10. K. Staras, G. Kemenes, P. R. Benjamin, Pattern-generating role for motoneurons in a rhythmically active neuronal network. *J Neurosci* **18**, 3669–88 (1998).
11. R. M. Rose, P. R. Benjamin, The Relationship of the Central Motor Pattern to the Feeding Cycle of Lymnaea Stagnalis. *Journal of Experimental Biology* **80**, 137–163 (1979).
12. R. M. Rose, P. R. Benjamin, Interneuronal Control of Feeding in the Pond Snail Lymnaea Stagnalis : II. The Interneuronal Mechanism Generating Feeding Cycles. *Journal of Experimental Biology* **92**, 203–228 (1981).
13. C. R. McCrohan, P. R. Benjamin, Synaptic Relationships of the Cerebral Giant Cells With Motoneurones in the Feeding System of Lymnaea Stagnalis. *Journal of Experimental Biology* **85**, 169–186 (1980).
14. C. J. Elliott, P. R. Benjamin, Interactions of pattern-generating interneurons controlling feeding in Lymnaea stagnalis. *J Neurophysiol* **54**, 1396–1411 (1985).
15. Vehovszky A, C. J. Elliott, Activation and reconfiguration of fictive feeding by the octopamine-containing modulatory OC interneurons in the snail Lymnaea. *J Neurophysiol* **86**, 792–808 (2001).
16. V. A. Straub, K. Staras, G. Kemenes, P. R. Benjamin, Endogenous and network properties of Lymnaea feeding central pattern generator interneurons. *J Neurophysiol* **88**, 1569–83 (2002).
